# Chronic Proton Pump Inhibitor Use and Glycemic Control in Older Adults with Type 2 Diabetes Mellitus: A Retrospective Cross-Sectional Study

**DOI:** 10.3390/biomedicines14102244

**Published:** 2026-10-04

**Authors:** Safiye Kübra Çetindağ Karatlı, Hatice Ergin, Merve Duygu Yurtbay, Meva Esra Doğan

**Affiliations:** 1 Department of Family Medicine, Ankara Yıldırım Beyazıt University, 06800 Ankara, Türkiye; 2 Department of Family Medicine, Ankara Bilkent City Hospital, 06800 Ankara, Türkiye; 3 Department of Internal Medicine, Ankara Bilkent City Hospital, 06800 Ankara, Türkiye; 4 Department of Intensive Care, Muğla Sıtkı Koçman Education and Research Hospital, 48000 Muğla, Türkiye

**Keywords:** proton pump inhibitors, type 2 diabetes mellitus, HbA1c, glycemic control, older adults, polypharmacy

## Abstract

**Background/Objectives:** Evidence regarding the effects of proton pump inhibitors (PPIs) on glucose metabolism is conflicting, and real-world data on the association between chronic PPI use and glycemic control in older adults with type 2 diabetes mellitus (T2DM) are limited. This study aimed to evaluate the association between chronic PPI use and glycemic control in patients with T2DM aged 65 years or older. **Methods:** This single-center, retrospective cross-sectional study included 462 T2DM patients attending the Family Medicine Outpatient Clinic of Ankara Bilkent City Hospital between April 2025 and April 2026. Patients with regular PPI use for ≥6 months before the index HbA1c were classified as chronic PPI users. Suboptimal glycemic control was defined as HbA1c ≥ 8.0%. Associations were evaluated using logistic regression for suboptimal control and linear regression for HbA1c level. **Results:** Of the 462 patients, 316 (68.4%) were chronic PPI users. Mean HbA1c (7.11 ± 1.21% vs. 6.23 ± 0.99%; *p* < 0.001) and fasting plasma glucose (148.70 ± 64.87 vs. 116.23 ± 43.54 mg/dL; *p* < 0.001) were higher among PPI users. In multivariable logistic regression, chronic PPI use was independently associated with suboptimal glycemic control (adjusted odds ratio [aOR] = 3.416; 95% CI: 1.572–7.422; *p* = 0.002). This association remained significant in a sensitivity model additionally adjusted for age, sex, body mass index (BMI), estimated glomerular filtration rate (eGFR), smoking status, diabetes duration, insulin use, metformin use, and heart failure/coronary artery disease (aOR = 3.834; 95% CI: 1.734–8.478; *p* = 0.001). In multivariable linear regression, chronic PPI use was independently associated with a 0.793-percentage-point higher HbA1c level (95% CI: 0.570–1.016; *p* < 0.001). **Conclusions:** Chronic PPI use was independently associated with higher HbA1c levels and suboptimal glycemic control in older adults with T2DM. Because of the observational design, causality cannot be established. Prospective studies accounting for PPI indication, agent-specific effects, dose, and duration of exposure are needed to clarify the nature of this association.

## 1. Introduction

Type 2 diabetes mellitus (T2DM) is a chronic metabolic disease with an increasing global prevalence and a substantial burden of morbidity and mortality. In older adults, glycemic control may be particularly challenging because of multimorbidity, polypharmacy, and long-term medication use; therefore, the effects of concomitant medications on glucose metabolism are clinically relevant [1,2,3]. Proton pump inhibitors (PPIs) are among the medications commonly used in older patients with T2DM because of gastroesophageal reflux disease, peptic ulcer disease, and the need for gastroprotection associated with antiplatelet or nonsteroidal anti-inflammatory drug use [3,4]. Long-term PPI use, sometimes extending beyond the original indication, underscores the importance of periodically reassessing treatment necessity and potential long-term effects, particularly in older adults in whom polypharmacy is common [5].

PPIs suppress gastric acid secretion by inhibiting the H+/K+-ATPase pump in gastric parietal cells and increase serum gastrin levels through negative feedback. Experimental data suggest that gastrin may promote pancreatic β-cell neogenesis and proliferation, enhance glucose-dependent insulin secretion, and exert incretin-like effects. PPI therapy has also been proposed to modify glucose homeostasis through effects on somatostatin levels and gastric emptying. These mechanisms provide a biological basis for the hypothesis that PPI use may improve glycemic control [6,7].

Earlier clinical studies reported that PPI use was associated with lower HbA1c levels and, in some studies, that this association remained independently significant for fasting plasma glucose [8,9].

However, subsequent studies have not consistently confirmed these findings. Observational studies evaluating long-term PPI use have failed to demonstrate a significant association between treatment duration and improvement in HbA1c. Although systematic reviews and meta-analyses have suggested that PPI therapy may provide modest short-term improvements in glycemic parameters, findings regarding long-term metabolic effects remain conflicting [10,11]. In addition, epidemiological studies have suggested that long-term PPI exposure may be associated with an increased risk of incident diabetes. Alterations in the gut microbiota, hypomagnesemia, reduced insulin-like growth factor-1 levels, and pregnane X receptor-mediated hepatic metabolic changes have been proposed as potential adverse mechanisms [12,13,14,15].

Most previous studies have focused on general adult populations or the risk of incident T2DM, whereas real-world data on the association between chronic PPI use and glycemic control in older patients with established T2DM and a high burden of comorbidity remain limited. This age group is of particular clinical relevance because multimorbidity, polypharmacy, and chronic PPI exposure are more common. Therefore, this study aimed to evaluate the association between chronic PPI use and glycemic control in patients with T2DM aged 65 years or older and to identify clinical and treatment-related factors independently associated with suboptimal glycemic control [5,16].

## 2. Materials and Methods

### 2.1. Study Design and Population

This single-center, retrospective cross-sectional study was conducted by reviewing the electronic medical records of patients with T2DM who attended the Family Medicine Outpatient Clinic of Ankara Bilkent City Hospital between April 2025 and April 2026. The study was approved by the ethics committee on 22 July 2026, and data collection was performed retrospectively thereafter.

### 2.2. Inclusion and Exclusion Criteria

Patients were eligible if they were aged 65 years or older, had a diagnosis of T2DM, attended the family medicine outpatient clinic, were receiving oral antidiabetic drugs (OADs) and/or insulin therapy, had at least one HbA1c and fasting plasma glucose measurement during the study period, and had medication-use information that could be reliably ascertained.

Patients with type 1 diabetes mellitus or secondary diabetes; those receiving active systemic corticosteroid therapy or active cancer treatment; those with end-stage kidney disease requiring dialysis or decompensated liver disease; those with a history of severe infection or acute hospitalization that could affect the index HbA1c assessment; and those with missing or incomplete data for the variables required for the planned analyses were excluded.

To avoid unstable estimates caused by very small cell counts in drug-specific analyses, antidiabetic drug groups with fewer than 10 users in the overall cohort (nateglinide/glinides and glitazones) were not included in the corresponding comparisons.

**Figure 1:**
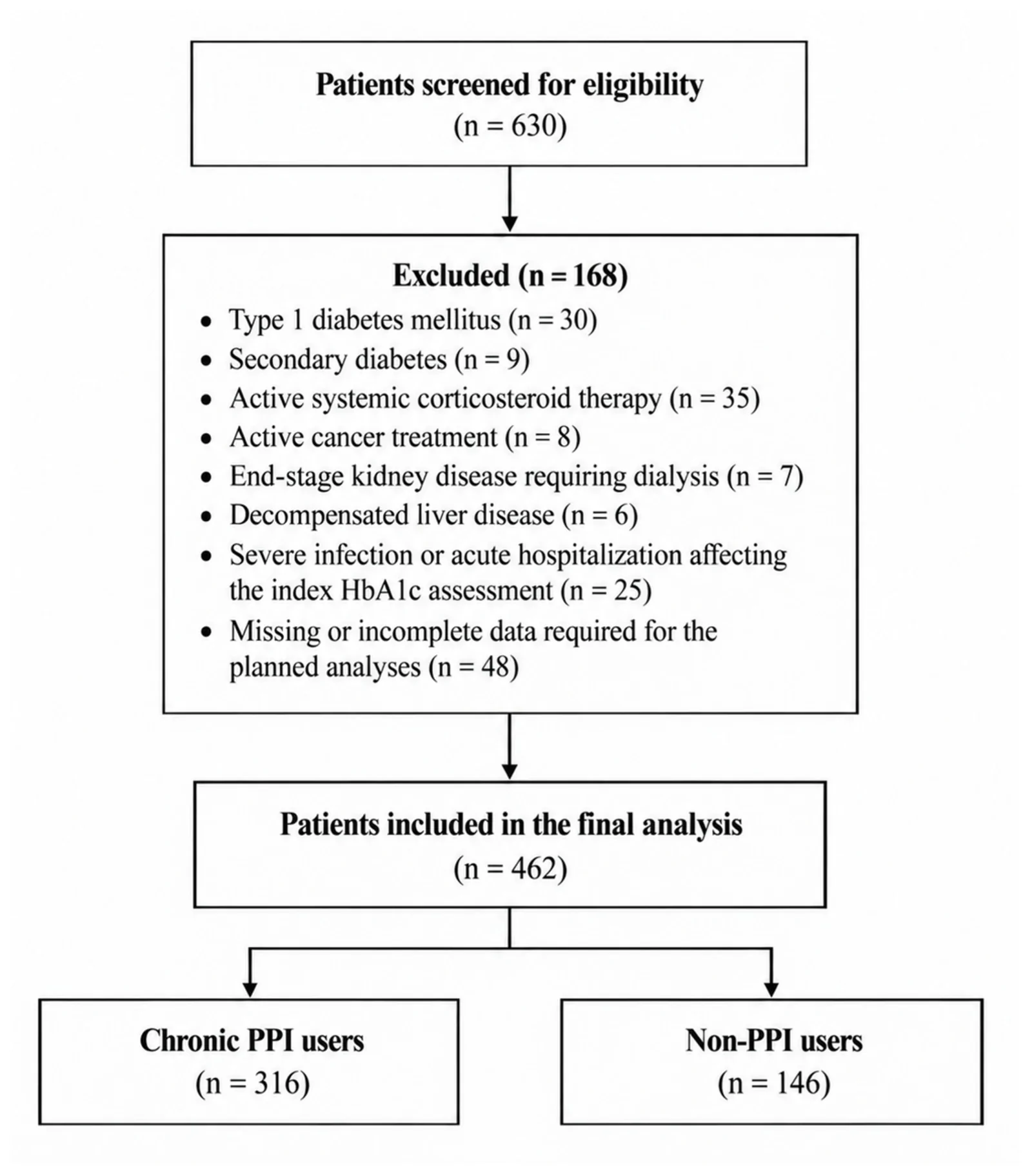
Flow diagram of patient screening, exclusion, and inclusion in the final study cohort.

A study flow diagram illustrating patient screening, exclusions, and final inclusion is shown in Figure 1.

### 2.3. Definition of PPI Exposure

For each patient, an HbA1c measurement for which PPI exposure during the preceding 6 months could be reliably assessed was designated as the index assessment. If a patient had more than one eligible HbA1c measurement during the study period, the most recent measurement for which PPI exposure during the preceding 6 months could be reliably verified was selected as the index HbA1c. To reduce misclassification of medication exposure, PPI and antidiabetic treatment use was verified by jointly reviewing electronic prescription/medication records and clinician-documented medical history and medication-use notes. Patients whose regular PPI use for at least 6 months before the index HbA1c measurement was confirmed by these sources were classified as chronic PPI users. The 6-month threshold was selected in accordance with current clinical definitions of chronic PPI use and to distinguish sustained exposure from short-term or intermittent use [17]. Patients with no PPI use during the 6 months preceding the index assessment were included in the non-PPI control group.

### 2.4. Clinical and Treatment Variables

Demographic and clinical variables included age, sex, diabetes duration, body mass index (BMI), estimated glomerular filtration rate (eGFR), smoking status, hypertension, hyperlipidemia, heart failure and/or coronary artery disease, chronic obstructive pulmonary disease (COPD)/asthma, thyroid disease, and neurological disease. Laboratory variables, including eGFR and hemoglobin, were obtained from the electronic medical records. Glycemic status was assessed using HbA1c and fasting plasma glucose levels. Among chronic PPI users, the specific PPI agent used at the index assessment was also recorded and categorized as pantoprazole, lansoprazole, omeprazole, rabeprazole, or esomeprazole.

Antidiabetic treatments, including metformin, gliclazide, linagliptin, vildagliptin, sitagliptin, dapagliflozin, empagliflozin, and insulin, were coded as independent binary variables. Because patients could receive more than one antidiabetic agent simultaneously, treatment groups were not considered mutually exclusive. Insulin therapy was analyzed as a binary variable (yes/no), irrespective of the insulin regimen used.

### 2.5. Outcomes

Suboptimal glycemic control was prespecified as HbA1c ≥ 8.0%, taking into account American Diabetes Association recommendations supporting individualized and less stringent glycemic targets in older adults with comorbidities. Accordingly, the association between PPI use and glycemic control was examined both using a clinically relevant categorical threshold and across the continuous distribution of HbA1c.

### 2.6. Statistical Analysis

Continuous variables were presented as mean ± standard deviation or median (interquartile range), according to their distribution, whereas categorical variables were presented as number and percentage. Comparisons between PPI users and nonusers were performed using the independent-samples t test or Mann–Whitney U test for continuous variables and Pearson’s chi-square test or Fisher’s exact test, as appropriate, for categorical variables. To quantify the magnitude of between-group differences independently of sample size, absolute standardized mean differences (SMDs) were additionally calculated for demographic, clinical, and treatment-related characteristics. An absolute SMD ≥ 0.10 was considered indicative of potentially meaningful between-group imbalance.

To identify factors associated with suboptimal glycemic control, defined as HbA1c ≥ 8.0%, univariable logistic regression analyses were first performed. Variables with *p* < 0.10 in univariable analyses were entered into the multivariable logistic regression model. Results were reported as odds ratios (ORs), adjusted odds ratios (aORs), and 95% confidence intervals (CIs). Model calibration was assessed using the Hosmer–Lemeshow goodness-of-fit test, and discrimination was evaluated using the area under the receiver operating characteristic curve (AUC).

In addition to the main model, a sensitivity multivariable logistic regression model was constructed to assess the robustness of the association between chronic PPI use and suboptimal glycemic control to potential confounding. Chronic PPI use was forced into the model as the primary exposure variable, together with age, sex, BMI, eGFR, smoking status, diabetes duration, insulin use, metformin use, and heart failure/coronary artery disease, which were prespecified on clinical grounds as relevant potential confounders. Thus, variables in the primary multivariable model were selected using statistical screening (*p* < 0.10 in univariable analysis), whereas the sensitivity model incorporated clinically relevant potential confounders irrespective of their univariable statistical significance.

For analyses in which HbA1c was treated as a continuous variable, univariable and multivariable linear regression analyses were performed. Variables with *p* < 0.10 in univariable analyses were entered into the multivariable model. Linear regression results were reported as unstandardized regression coefficients (B) with 95% CIs. Model fit was assessed using the coefficient of determination (R^2^) and adjusted R^2^. Multicollinearity was evaluated using variance inflation factors (VIFs).

Because patients with missing or incomplete data for variables required in the planned analyses were excluded, the regression analyses were performed on complete cases.

All tests were two-sided, and *p* < 0.05 was considered statistically significant. Statistical analyses were performed using SPSS software (version 29.0; IBM Corp., Armonk, NY, USA).

### 2.7. Ethics Approval

The study was approved by the Ankara Bilkent City Hospital Scientific and Ethical Evaluation Board for Medical Research (TABED) on 22 July 2026 (Decision No. 2-26-2528) and was conducted in accordance with the principles of the Declaration of Helsinki. Following ethics approval, data were retrospectively extracted from the medical records of patients who attended the clinic between April 2025 and April 2026.

## 3. Results

### 3.1. Patient Characteristics

A total of 462 patients with T2DM who attended the Family Medicine Outpatient Clinic of Ankara Bilkent City Hospital between April 2025 and April 2026 were included. Of these, 316 (68.4%) were chronic PPI users and 146 (31.6%) were non-users.

Mean age was similar between PPI users and non-users (81.80 ± 9.17 vs. 81.68 ± 11.07 years, respectively; *p* = 0.909), and there was no significant between-group difference in diabetes duration (14.41 ± 6.60 vs. 15.48 ± 6.81 years; *p* = 0.115). The proportion of women was higher among PPI users, although the difference did not reach statistical significance (73.4% vs. 65.1%; *p* = 0.067). BMI, eGFR, and smoking status were also comparable between the groups, with no statistically significant differences observed (all *p* > 0.05). Mean hemoglobin levels were comparable between PPI users and non-PPI users (11.61 ± 1.90 vs. 11.38 ± 1.98 g/dL, respectively; *p* = 0.219).

With respect to glycemic parameters, both mean HbA1c (7.11 ± 1.21% vs. 6.23 ± 0.99%; *p* < 0.001) and fasting plasma glucose (148.70 ± 64.87 vs. 116.23 ± 43.54 mg/dL; *p* < 0.001) were significantly higher among PPI users than among non-users. Suboptimal glycemic control (HbA1c ≥ 8.0%) was also more frequent among chronic PPI users than among non-PPI users (18.7% [59/316] vs. 5.5% [8/146], respectively; *p* < 0.001).

The prevalence of hypertension, hyperlipidemia, COPD/asthma, thyroid disease, and neurological disease was similar between groups. In contrast, heart failure and/or coronary artery disease was more frequent among PPI users than among non-users (48.4% vs. 38.4%; *p* = 0.043) (Table 1). Assessment of absolute SMDs demonstrated potentially meaningful between-group imbalance in several baseline characteristics, including female sex (SMD = 0.181), diabetes duration (SMD = 0.160), heart failure/coronary artery disease (SMD = 0.203), COPD/asthma (SMD = 0.173), and neurological disease (SMD = 0.140).

### 3.2. Distribution of Antidiabetic Treatments

Metformin use was significantly more common among non-PPI users than among PPI users (62.3% vs. 50.3%; *p* = 0.016). Insulin use was markedly more frequent among PPI users (42.7% vs. 24.7%; *p* < 0.001). Vildagliptin use was also more frequent in the PPI group (6.6% vs. 2.1%; *p* = 0.039). No statistically significant between-group differences were observed for gliclazide, linagliptin, sitagliptin, dapagliflozin, or empagliflozin use (Table 2a). Absolute SMDs demonstrated notable imbalance in insulin use (SMD = 0.388), metformin use (SMD = 0.244), and vildagliptin use (SMD = 0.222), with a smaller imbalance in dapagliflozin use (SMD = 0.109).

Because a patient could receive more than one antidiabetic medication simultaneously, treatment categories were not mutually exclusive, and percentages may sum to more than 100%. PPI: proton pump inhibitor.

Among chronic PPI users, pantoprazole was the most frequently used agent (31.6%), followed by omeprazole (25.6%), esomeprazole (15.5%), lansoprazole (15.2%), and rabeprazole (12.0%) (Table 2b).

### 3.3. Factors Associated with Suboptimal Glycemic Control

In univariable logistic regression analysis, chronic PPI use was strongly associated with suboptimal glycemic control (HbA1c ≥ 8.0%) (OR = 3.960; 95% CI: 1.839–8.526; *p* < 0.001). Insulin use was also associated with increased odds of suboptimal glycemic control (OR = 2.775; 95% CI: 1.636–4.708; *p* < 0.001). BMI, eGFR, smoking status, and specific PPI type were not significantly associated with suboptimal glycemic control in univariable analyses (all *p* > 0.05). Likewise, no statistically significant associations were observed for the other demographic characteristics, comorbidities, or antidiabetic treatments evaluated (Table 3).

In the multivariable model including variables with *p* < 0.10 in univariable analyses, chronic PPI use and insulin use remained independently associated with suboptimal glycemic control after adjustment. Chronic PPI use was associated with higher odds of suboptimal glycemic control (aOR = 3.416; 95% CI: 1.572–7.422; *p* = 0.002), whereas insulin use was also associated with higher odds of suboptimal glycemic control (aOR = 2.409; 95% CI: 1.407–4.125; *p* = 0.001) (Table 3). Given the cross-sectional design, these findings should be interpreted as associations and not as evidence of increased risk or causality. The logistic regression model showed adequate calibration according to the Hosmer–Lemeshow goodness-of-fit test (χ^2^ = 3.404, df = 2, *p* = 0.182) and modest discrimination, with an area under the ROC curve (AUC) of 0.639 (95% CI: 0.572–0.707; *p* < 0.001). There were 67 events of suboptimal glycemic control in the cohort. The primary multivariable logistic regression model included two predictors, corresponding to approximately 33.5 events per predictor.

In an additional multivariable logistic regression sensitivity analysis including clinically relevant potential confounders (age, sex, BMI, eGFR, smoking status, diabetes duration, insulin use, metformin use, and heart failure/coronary artery disease), chronic PPI use remained independently associated with suboptimal glycemic control (aOR = 3.834; 95% CI: 1.734–8.478; *p* = 0.001). Insulin use also remained independently associated with suboptimal glycemic control (aOR = 2.394; 95% CI: 1.309–4.379; *p* = 0.005), whereas metformin use was not independently associated with the outcome (aOR = 1.458; 95% CI: 0.789–2.691; *p* = 0.229). Age, sex, BMI, eGFR, smoking status, diabetes duration, and heart failure/coronary artery disease were not significantly associated with the outcome in this sensitivity model (all *p* > 0.05) (Supplementary Table S1).

### 3.4. Factors Associated with HbA1c Level

In univariable linear regression analysis, chronic PPI use was significantly associated with higher HbA1c levels (B = 0.883; 95% CI: 0.657–1.109; *p* < 0.001). Each 1-year increase in diabetes duration was associated with a 0.025-percentage-point increase in HbA1c (B = 0.025; 95% CI: 0.008–0.041; *p* = 0.004), while insulin use was associated with a 0.720-percentage-point higher HbA1c level (B = 0.720; 95% CI: 0.499–0.942; *p* < 0.001). BMI, eGFR, and smoking status were not significantly associated with HbA1c levels in univariable analyses (all *p* > 0.05). Similarly, no significant associations were observed for the other evaluated demographic characteristics, comorbidities, or antidiabetic treatments, except for variables subsequently included in the multivariable model (Table 4).

In multivariable linear regression analysis, chronic PPI use remained independently associated with a 0.793-percentage-point higher HbA1c level (B = 0.793; 95% CI: 0.570–1.016; *p* < 0.001). Diabetes duration (B = 0.018; 95% CI: 0.003–0.034; *p* = 0.022) and insulin use (B = 0.517; 95% CI: 0.299–0.734; *p* < 0.001) were also independently associated with HbA1c level. Age, heart failure/coronary artery disease, sitagliptin use, and empagliflozin use did not reach statistical significance in the multivariable model (Table 4). The multivariable linear regression model explained 19.5% of the variance in HbA1c (R^2^ = 0.195; adjusted R^2^ = 0.182). Variance inflation factors ranged from 1.027 to 1.089, indicating no evidence of problematic multicollinearity.

## 4. Discussion

In this study, chronic PPI use was significantly and independently associated with suboptimal glycemic control in patients with T2DM aged 65 years or older. Chronic PPI users had higher HbA1c and fasting plasma glucose levels, and the increased odds of suboptimal glycemic control persisted after adjustment for clinically relevant potential confounders. Similarly, in multivariable linear regression with HbA1c analyzed as a continuous variable, chronic PPI use remained independently associated with higher HbA1c levels. These findings suggest a potentially unfavorable association between long-term PPI exposure and glycemic control in older adults with T2DM. The adjusted 0.793-percentage-point difference in HbA1c associated with chronic PPI use may be clinically relevant in magnitude. However, because this estimate was derived from a cross-sectional observational analysis and residual confounding cannot be excluded, it should not be interpreted as a treatment effect attributable to PPI exposure.

Evidence regarding the relationship between PPI use and glucose metabolism remains conflicting. Some studies have reported that PPI use may be associated with lower HbA1c and fasting plasma glucose levels, with this potential benefit attributed to hypergastrinemia-related β-cell proliferation and increased insulin secretion [7,8,9]. In contrast, other observational studies and systematic evaluations have not demonstrated consistent glycemic improvement with long-term use [10,11]. Thus, the available literature does not consistently support a beneficial glycemic effect of PPI therapy. More recent studies have also reported adverse metabolic associations, including increases in HbA1c and fasting glucose or a higher risk of incident T2DM. Therefore, the direction of the association observed in our study is consistent with part of the more recent literature, although differences in study populations, exposure definitions, diabetes severity, and duration of PPI use may account for the heterogeneity across studies. These discrepancies may reflect differences in PPI exposure duration, patient characteristics, and baseline metabolic status, as well as mechanisms associated with prolonged use, including hypomagnesemia, insulin resistance, lower IGF-1 levels, alterations in the gut microbiota, and PXR-mediated metabolic effects [14,15]. Thus, the effects of PPIs on glucose metabolism may differ according to the duration of exposure. Although total PPI exposure duration could not be evaluated in detail in our study, longer-term medication use may be more common in this older population and may have contributed to the observed unfavorable glycemic association. These proposed mechanisms were not directly assessed in the present study and should therefore be regarded as hypothesis-generating explanations rather than demonstrated biological pathways.

More recent epidemiological evidence also suggests that chronic PPI use may be associated with adverse metabolic outcomes. In prospective cohorts, regular PPI use has been associated with a 24–69% increase in the risk of incident T2DM, with higher risks reported with longer treatment duration and higher doses [13,14]. Meta-analyses have likewise demonstrated a positive association between PPI exposure and T2DM risk; however, substantial between-study heterogeneity warrants cautious interpretation [12,15]. Similarly, a prospective study of individuals without diabetes reported increases in HbA1c and fasting glucose over time, while another large population analysis found that the association between PPI use and T2DM persisted after extensive covariate adjustment and propensity score matching, with comparable metabolic signals also observed in pharmacovigilance analyses [18,19]. However, these studies primarily evaluated the development of incident T2DM in populations without established diabetes and therefore address a different clinical outcome from the present study, which examined glycemic control among older adults with pre-existing T2DM.

The clinical profile of the study population should be considered when interpreting these findings. Insulin use and heart failure/coronary artery disease were more frequent among PPI users. This pattern suggests that PPI use may partly serve as a marker of greater comorbidity and treatment burden and raises the possibility of confounding by indication. The association of insulin use with both suboptimal glycemic control and higher HbA1c likely reflects more advanced or longer-standing diabetes rather than an adverse effect of insulin itself. Nevertheless, chronic PPI use remained independently associated with suboptimal glycemic control even in the sensitivity model, additionally accounting for age, sex, BMI, eGFR, smoking status, diabetes duration, insulin use, metformin use, and cardiovascular comorbidity. Although adjustment for clinically relevant covariates reduced the potential impact of measured confounding, residual confounding remains possible because important factors such as the indication for PPI therapy, PPI dose, cumulative duration of use, treatment adherence, and detailed measures of diabetes severity were not available. Therefore, the observed association should be interpreted cautiously. In addition, the more extensively adjusted sensitivity model included a larger number of covariates relative to the number of outcome events and should therefore be interpreted primarily as a robustness analysis rather than as a definitive predictive model. These findings may suggest that the observed association is not clearly driven by a single PPI agent; however, the relatively small numbers within individual PPI subgroups limit firm agent-specific conclusions.

These findings may have clinical relevance in older adults with T2DM, among whom multimorbidity and polypharmacy are common. The indication for and continued need for chronic PPI therapy should therefore be periodically reassessed in accordance with established clinical indications [4,5]. However, given the observational nature of our study, the present findings do not support changing or discontinuing PPI therapy solely on the basis of glycemic control. Rather, they highlight the importance of medication review and regular monitoring of glycemic status in older adults receiving long-term PPI therapy.

A key contribution of this study is the evaluation of the association between chronic PPI use and glycemic control in a real-world population of older adults with established T2DM. Whereas much of the previous literature has focused on incident T2DM, data on HbA1c and suboptimal glycemic control among older patients with existing diabetes remain limited. Additional strengths include the real-world cohort of 462 patients, the definition of chronic PPI use as regular exposure for at least 6 months before the index HbA1c measurement, assessment of the association using both categorical and continuous HbA1c outcomes, and persistence of the association after additional adjustment for clinically relevant potential confounders. Together, these features support the internal consistency of the findings and contribute to an area in which evidence remains limited.

Several limitations should be acknowledged. First, the retrospective cross-sectional design precludes causal inference and does not completely exclude the possibility of reverse causation. In addition, the single-center family medicine outpatient setting may introduce selection bias and may limit the generalizability of the findings to other clinical settings or populations. The study population was characterized by advanced age, a relatively low mean BMI, and a high prevalence of chronic PPI use. The advanced age is consistent with the study’s intentional focus on adults aged 65 years or older. Nevertheless, these characteristics may reflect the specific case mix of this hospital-based outpatient population and may further limit the generalizability of the findings to broader populations of older adults with T2DM. Second, several potentially relevant confounders, including waist circumference, dietary characteristics, physical activity, frailty, socioeconomic factors, detailed measures of diabetes severity, antiplatelet or anticoagulant therapy, and total medication burden, could not be reliably obtained from the retrospective records. Ferritin and vitamin B12 levels were not consistently available in the retrospective records; therefore, potential effects of iron or vitamin B12 deficiency on HbA1c measurement could not be fully evaluated. Furthermore, chronic PPI use may partly reflect greater multimorbidity, medication burden, or healthcare utilization rather than a direct effect of PPI exposure itself. Although BMI, eGFR, smoking status, and major clinical comorbidities were incorporated into the sensitivity analysis, residual and unmeasured confounding cannot be excluded. Moreover, the indication for PPI therapy, PPI dose, adherence, and total cumulative duration of exposure could not be evaluated in detail. Consequently, dose–response and indication-specific associations could not be evaluated, which limits interpretation of the exposure–outcome relationship. Although exploratory analyses were performed according to individual PPI agent, subgroup sizes were relatively limited and these analyses were not powered to establish agent-specific differences. Finally, because a single index HbA1c measurement was used, within-patient changes in HbA1c before and after PPI initiation could not be assessed.

## 5. Conclusions

Chronic PPI use was independently associated with higher HbA1c levels and suboptimal glycemic control in older adults with T2DM. These findings suggest that chronic PPI exposure may be associated with glycemic control in older individuals with established diabetes. However, because of the observational design, a causal relationship cannot be established. Prospective studies incorporating detailed assessment of PPI indication, agent-specific effects, dose, cumulative exposure duration, treatment adherence, and serial HbA1c measurements are needed to clarify the nature of this association.

## Figures and Tables

**Table 1:** Demographic and clinical characteristics according to chronic PPI use.

Variable	Non-PPI Users (*n* = 146)	PPI Users (*n* = 316)	*p*	Absolute SMD
Age, years, mean ± SD	81.68 ± 11.07	81.80 ± 9.17	0.909	0.012
Female sex, n (%)	95 (65.1)	232 (73.4)	0.067	0.181
Diabetes duration, years, mean ± SD	15.48 ± 6.81	14.41 ± 6.60	0.115	0.160
BMI, kg/m^2^, mean ± SD	21.29 ± 5.08	21.47 ± 5.11	0.719	0.035
eGFR, mL/min/1.73 m^2^, mean ± SD	64.25 ± 14.30	62.72 ± 15.02	0.302	0.104
Hemoglobin, g/dL, mean ± SD	11.38 ± 1.98	11.61 ± 1.90	0.219	0.123
Smoking, n (%)	46 (31.5)	115 (36.4)	0.306	0.104
HbA1c, %, mean ± SD	6.23 ± 0.99	7.11 ± 1.21	<0.001	0.796
Fasting plasma glucose, mg/dL, mean ± SD	116.23 ± 43.54	148.70 ± 64.87	<0.001	0.588
Hypertension, n (%)	128 (87.7)	279 (88.3)	0.848	0.018
Heart failure/coronary artery disease, n (%)	56 (38.4)	153 (48.4)	0.043	0.203
Hyperlipidemia, n (%)	90 (61.6)	206 (65.2)	0.460	0.075
COPD/asthma, n (%)	11 (7.5)	40 (12.7)	0.102	0.173
Thyroid disease, n (%)	23 (15.8)	49 (15.5)	0.946	0.008
Neurological disease, n (%)	48 (32.9)	125 (39.6)	0.168	0.140
Suboptimal glycemic control (HbA1c ≥ 8.0%), n (%)	8 (5.5)	59 (18.7)	<0.001	0.413

Table note: Continuous variables are presented as mean ± standard deviation and categorical variables as n (%). Continuous variables were compared using the independent-samples t test, and categorical variables using Pearson’s chi-square test, as appropriate. BMI: body mass index; eGFR: estimated glomerular filtration rate; PPI: proton pump inhibitor; SD: standard deviation; HbA1c: glycated hemoglobin; COPD: chronic obstructive pulmonary disease. SMD: standardized mean difference. Absolute SMD values ≥ 0.10 were considered indicative of potentially meaningful between-group imbalance.

**Table 2:** (**a**) Distribution of antidiabetic treatments according to chronic PPI use. (**b**) Distribution of specific PPI agents among chronic PPI users.

(a)				
Antidiabetic Treatment	Non-PPI Users (*n* = 146)	PPI Users (*n* = 316)	*p*	Absolute SMD
Metformin, n (%)	91 (62.3)	159 (50.3)	0.016	0.244
Gliclazide, n (%)	15 (10.3)	39 (12.3)	0.520	0.063
Linagliptin, n (%)	38 (26.0)	79 (25.0)	0.813	0.023
Vildagliptin, n (%)	3 (2.1)	21 (6.6)	0.039	0.222
Sitagliptin, n (%)	14 (9.6)	38 (12.0)	0.441	0.077
Dapagliflozin, n (%)	10 (6.8)	31 (9.8)	0.298	0.109
Empagliflozin, n (%)	10 (6.8)	22 (7.0)	0.965	0.008
Insulin use, any regimen, n (%)	36 (24.7)	135 (42.7)	<0.001	0.388
(**b**)				
**PPI Agent**	***n***		**%**	
Pantoprazole	100		31.6	
Lansoprazole	48		15.2	
Omeprazole	81		25.6	
Rabeprazole	38		12.0	
Esomeprazole	49		15.5	
Total	316		100.0	

(**a**) Table Note: SMD: standardized mean difference. Absolute SMD values ≥ 0.10 were considered indicative of potentially meaningful between-group imbalance. (**b**) Table note: Percentages were calculated among chronic PPI users only (n = 316). PPI: proton pump inhibitor.

**Table 3:** Univariable and multivariable logistic regression analyses of factors associated with suboptimal glycemic control (HbA1c ≥ 8.0%).

Variable	Univariable OR	95% CI	*p*	Adjusted OR	95% CI	*p*
Chronic PPI use	3.960	1.839–8.526	<0.001	3.416	1.572–7.422	0.002
PPI type, overall	—	—	0.139	—	—	—
Lansoprazole vs. pantoprazole	0.350	0.097–1.265	0.109	—	—	—
Omeprazole vs. pantoprazole	1.500	0.710–3.171	0.288	—	—	—
Rabeprazole vs. pantoprazole	1.875	0.764–4.604	0.170	—	—	—
Esomeprazole vs. pantoprazole	1.346	0.560–3.235	0.506	—	—	—
Sex	1.374	0.753–2.508	0.300	—	—	—
Age, per 1-year increase	1.023	0.992–1.055	0.141	—	—	—
Diabetes duration, per 1-year increase	1.028	0.991–1.067	0.145	—	—	—
BMI, per 1 kg/m^2^ increase	0.957	0.897–1.021	0.188	—	—	—
eGFR, per 1 mL/min/1.73 m^2^ increase	1.015	0.997–1.034	0.110	—	—	—
Smoking	0.767	0.437–1.345	0.354	—	—	—
Hypertension	1.440	0.591–3.507	0.422	—	—	—
Hyperlipidemia	1.173	0.677–2.033	0.568	—	—	—
Heart failure/coronary artery disease	1.490	0.886–2.506	0.132	—	—	—
COPD/asthma	0.931	0.400–2.163	0.867	—	—	—
Thyroid disease	0.818	0.385–1.735	0.600	—	—	—
Neurological disease	1.151	0.678–1.954	0.602	—	—	—
Insulin use	2.775	1.636–4.708	<0.001	2.409	1.407–4.125	0.001
Metformin use	0.796	0.474–1.337	0.389	—	—	—
Gliclazide use	1.400	0.667–2.938	0.374	—	—	—
Linagliptin use	1.003	0.553–1.819	0.992	—	—	—
Vildagliptin use	1.596	0.575–4.431	0.370	—	—	—
Sitagliptin use	1.475	0.701–3.104	0.306	—	—	—
Dapagliflozin use	0.804	0.304–2.129	0.661	—	—	—
Empagliflozin use	1.396	0.552–3.531	0.481	—	—	—

The dependent variable was suboptimal glycemic control (HbA1c ≥ 8.0%). Variables with *p* < 0.10 in univariable analyses were entered into the multivariable logistic regression model. The final model included chronic PPI use and insulin use. OR: odds ratio; CI: confidence interval; PPI: proton pump inhibitor; COPD: chronic obstructive pulmonary disease.

**Table 4:** Univariable and multivariable linear regression analyses of factors associated with HbA1c level.

Variable	Univariable B	95% CI	*p*	Adjusted B	95% CI	*p*
Chronic PPI use	0.883	0.657–1.109	<0.001	0.793	0.570–1.016	<0.001
Sex	−0.093	−0.339–0.152	0.454	—	—	—
Age, per 1-year increase	0.010	−0.001–0.022	0.080	0.009	−0.002–0.019	0.110
Diabetes duration, per 1-year increase	0.025	0.008–0.041	0.004	0.018	0.003–0.034	0.022
BMI, per 1 kg/m^2^ increase	−0.002	−0.024–0.020	0.843	—	—	—
eGFR, per 1 mL/min/1.73 m^2^ increase	0.005	−0.002–0.013	0.169	—	—	—
Smoking	−0.189	−0.422–0.045	0.113	—	—	—
Hypertension	0.038	−0.307–0.383	0.828	—	—	—
Hyperlipidemia	0.186	−0.046–0.418	0.116	—	—	—
Heart failure/coronary artery disease	0.191	−0.032–0.415	0.093	0.082	−0.123–0.287	0.433
COPD/asthma	−0.051	−0.407–0.305	0.779	—	—	—
Thyroid disease	0.005	−0.303–0.312	0.976	—	—	—
Neurological disease	0.035	−0.195–0.266	0.765	—	—	—
Insulin use	0.720	0.499–0.942	<0.001	0.517	0.299–0.734	<0.001
Metformin use	−0.136	−0.359–0.088	0.233	—	—	—
Gliclazide use	0.126	−0.222–0.473	0.478	—	—	—
Linagliptin use	0.158	−0.099–0.414	0.227	—	—	—
Vildagliptin use	0.351	−0.150–0.853	0.170	—	—	—
Sitagliptin use	0.309	−0.043–0.661	0.085	0.274	−0.051–0.599	0.098
Dapagliflozin use	0.084	−0.309–0.476	0.676	—	—	—
Empagliflozin use	0.414	−0.024–0.852	0.064	0.364	−0.038–0.767	0.076

The dependent variable was continuous HbA1c level. Variables with *p* < 0.10 in univariable analyses were entered into the multivariable linear regression model. Accordingly, PPI use, age, diabetes duration, heart failure/coronary artery disease, insulin use, sitagliptin use, and empagliflozin use were included in the multivariable model. B denotes the unstandardized regression coefficient; CI, confidence interval.

## Data Availability

The datasets are available from the corresponding author on reasonable request.

## Supplementary Materials

The following supporting information can be downloaded at: https://www.mdpi.com/article/10.3390/biomedicines14102244/s1, Table S1: Multivariable logistic regression analysis of suboptimal glycemic control.

## Abbreviations

The following abbreviations are used in this manuscript:

T2DM	Type 2 diabetes mellitus
PPI	Proton pump inhibitors
OAD	Oral antidiabetic drug
COPD	Chronic obstructive pulmonary disease
BMI	Body mass index
eGFR	Estimated glomerular filtration rate

## References

[1] International Diabetes Federation (2021). IDF Diabetes Atlas.

[2] Liu M.Z., He H.Y., Luo J.Q., He F.Z., Chen Z.R., Liu Y.P., Xiang D.X., Zhou H.H., Zhang W. (2018). Drug-induced hyperglycaemia and diabetes: Pharmacogenomics perspectives. Arch. Pharm. Res..

[3] Oktora M.P., Denig P., Bos J.H.J., Schuiling-Veninga C.C.M., Hak E. (2021). Trends in polypharmacy and potentially inappropriate medication (PIM) in older and middle-aged people treated for diabetes. Br. J. Clin. Pharmacol..

[4] Hálfdánarson Ó.Ö., Pottegård A., Björnsson E.S., Lund S.H., Ogmundsdottir M.H., Steingrímsson E., Ogmundsdottir H.M., Zoega H. (2018). Proton-pump inhibitors among adults: A nationwide drug-utilization study. Ther. Adv. Gastroenterol..

[5] Andrawes M., Andrawes W., Das A., Siau K. (2025). Proton Pump Inhibitors (PPIs)—An evidence-based review of indications, efficacy, harms, and deprescribing. Medicina.

[6] Sanaka M., Yamamoto T., Kuyama Y. (2010). Effects of proton pump inhibitors on gastric emptying: A systematic review. Dig. Dis. Sci..

[7] Boj-Carceller D. (2013). Proton pump inhibitors: Impact on glucose metabolism. Endocrine.

[8] Crouch M.A., Mefford I.N., Wade E.U. (2012). Proton pump inhibitor therapy associated with lower glycosylated hemoglobin levels in type 2 diabetes. J. Am. Board Fam. Med..

[9] Barchetta I., Guglielmi C., Bertoccini L., Calella D., Manfrini S., Secchi C., Pozzilli P., Cavallo M.G. (2015). Therapy with proton pump inhibitors in patients with type 2 diabetes is independently associated with improved glycometabolic control. Acta Diabetol..

[10] Trang A., Bushman J., Halalau A. (2021). Effect of long-term proton pump inhibitor use on glycemic control in patients with type 2 diabetes mellitus. J. Diabetes Res..

[11] Peng C.C.H., Tu Y.K., Lee G.Y., Chang R.H.E., Huang Y., Bukhari K., Tsai Y.C., Fu Y., Huang H.K., Munir K.M. (2021). Effects of proton pump inhibitors on glycemic control and incident diabetes: A systematic review and meta-analysis. J. Clin. Endocrinol. Metab..

[12] Guo Y.R., Liu X.M., Wang G.X. (2023). Exposure to proton pump inhibitors and risk of diabetes: A systematic review and meta-analysis. World J. Diabetes.

[13] Yuan J., He Q., Nguyen L.H., Wong M.C.S., Huang J., Yu Y., Xia B., Tang Y., He Y., Zhang C. (2021). Regular use of proton pump inhibitors and risk of type 2 diabetes: Results from three prospective cohort studies. Gut.

[14] Czarniak P., Ahmadizar F., Hughes J., Parsons R., Kavousi M., Ikram M.A., Stricker B.H. (2022). Proton pump inhibitors are associated with incident type 2 diabetes mellitus in a prospective population-based cohort study. Br. J. Clin. Pharmacol..

[15] Chenchula S., Sharma P., Ghanta M.K., Amerneni K.C., Rajakarunakaran P., Saggurthi P., Chandra M.B., Gupta R., Chavan M. (2024). Association and mechanisms of proton pump inhibitors use with type-2 diabetes mellitus incidence in adults: A systematic review and meta-analysis. Curr. Diabetes Rev..

[16] Bellary S., Kyrou I., Brown J.E., Bailey C.J. (2021). Type 2 diabetes mellitus in older adults: Clinical considerations and management. Nat. Rev. Endocrinol..

[17] Desai M., Ruan W., Thosani N.C., Amaris M., Scott J.S., Saeed A., Abu Dayyeh B., Canto M.I., Abidi W., Alipour O. (2025). American Society for Gastrointestinal Endoscopy guideline on the diagnosis and management of GERD: Summary and recommendations. Gastrointest. Endosc..

[18] Rangshoo A., Parveen S., Shah N.A., Qurieshi M.A., Iqbal U., Immad S. (2025). Long-term use of proton pump inhibitors and its impact on glucose homeostasis: Results of a prospective observational study. Int. J. Basic Clin. Pharmacol..

[19] Liang C., Zhang Y. (2025). Proton pump inhibitors use and risk of type 2 diabetes mellitus: Correlation analysis, prediction model construction, and key genes identification. Front. Pharmacol..

