# Supplementary material for: Chronic Proton Pump Inhibitor Use and Glycemic Control in Older Adults with Type 2 Diabetes Mellitus: A Retrospective Cross-Sectional Study
Source: Biomedicines. 2026 Oct 4;14(10):2244. doi: 10.3390/biomedicines14102244 (PMC13701455; doi:10.3390/biomedicines14102244)
Supplement: Supplementary file 1 [file biomedicines-14-02244-s001.zip › biomedicines-4598726-supplementary.pdf]

Supplementary Table S1. Multivariable logistic regression analysis of suboptimal glycemic control

Variable	Adjusted OR	95% CI	p
Chronic PPI use	3.834	1.734–8.478	0.001
Sex	1.225	0.651–2.305	0.528
Age	1.028	0.991–1.066	0.143
Insulin use	2.394	1.309–4.379	0.005
eGFR	1.014	0.995–1.034	0.149
BMI	0.953	0.889–1.021	0.169
Smoking	0.738	0.410–1.327	0.310
Diabetes duration	1.019	0.977–1.062	0.376
Heart failure/CAD	1.389	0.805–2.395	0.237
Metformin use	1.458	0.789–2.691	0.229

Note: The dependent variable was defined as suboptimal glycemic control (HbA1c ≥8.0%). The sensitivity multivariable model included chronic PPI use, age, sex, BMI, eGFR, smoking status, diabetes duration, insulin use, metformin use, and heart failure/coronary artery disease as clinically relevant potential confounders.
